# The Big Five personality traits and regularity of lifetime dental visit attendance: evidence of the Survey of Health, Ageing, and Retirement in Europe (SHARE)

**DOI:** 10.1007/s40520-021-02051-2

**Published:** 2021-12-28

**Authors:** Ghazal Aarabi, Carolin Walther, Kübra Kaymaz, Kristin Spinler, Elzbieta Buczak-Stec, Hans-Helmut König, André Hajek

**Affiliations:** 1 Department of Periodontics, Preventive and Restorative Dentistry, University Medical Center Hamburg-Eppendorf, Hamburg, Germany; 2 Institute of Medical Sociology, Center for Psychosocial Medicine, University Medical Center Hamburg-Eppendorf, Hamburg, Germany; 3 Department of Health Economics and Health Services Research, University Medical Center Hamburg-Eppendorf, Hamburg Center for Health Economics, Martinistr. 52, 20246 Hamburg, Germany

**Keywords:** Dental service, Dental visits, Dental care use, Dental care utilization, Oral health services, Quality of life, SHARE, Personality, Extraversion, Conscientiousness, Openness to experience, Agreeableness, Neuroticism, Emotional stability, Big Five

## Abstract

**Background:**

Regular dental visits are essential for the prevention, early detection and treatment of worldwide highly prevalent oral diseases. Personality traits were previously associated with treatment compliance, medication adherence and regular doctor visits, however, the link between personality traits and regular dental visit attendance remains largely unexplored. Thus, the objective of this study is to clarify this link.

**Methods:**

Data (wave 7) of the Survey of Health, Ageing and Retirement in Europe (SHARE) were used, focusing on Germany (*n *= 2822). Personality was assessed using the 10-item Big Five Inventory (BFI-10). Regular dental visits were assessed. Multiple logistic regressions were used, adjusting for various covariates.

**Results:**

Majority of the participants (84%) reported to attend regular dental visits during lifetime. Regularity of lifetime dental visit attendance was positively and significantly associated with increased extraversion [OR 1.13, 95% CI (1.01–1.26)], increased conscientiousness [OR 1.26, 95% CI (1.10–1.44)], and increased openness to experience [OR 1.12, 95% CI (1.01–1.26)]. However, there was a lack of association with agreeableness and neuroticism. Moreover, the outcome measure was positively associated with younger age, being female, born in Germany, being married, higher education, being retired (compared to being homemaker), whereas it was not associated with obesity or chronic diseases.

**Conclusions:**

Identification of personality traits that are associated with regular dental visits can support prevention, screening and clinical management of oral diseases. Further research in this field may facilitate the development and increase the incorporation of individualized concepts to enhance patient compliance and attendance, and thus the provision of oral and dental care services.

**Supplementary Information:**

The online version contains supplementary material available at 10.1007/s40520-021-02051-2.

## Introduction

More than half of the global population lacks essential health services [1], including oral health services. Need of appropriate oral health services is a significant matter, since 3.58 billion people of the global population were affected by oral diseases as of 2016, which include untreated dental caries, severe periodontitis and oral cancer [2]. Mostly, the disadvantaged members of the society are affected by oral diseases, where access to oral health services is often non-existent or limited [3]. However—beyond the existing need in the majority of the global population—the dental service use still remains low even in high-income countries [4]. In Germany, basic dental services are part of the statutory health insurance, though out-of-pocket payments for dental prosthetics are common [5].

Socioeconomic status [6, 7], migration background [8], level of education [9] are significant determinants of dental service use and thereof oral healthcare utilization [10, 11]. Yet, further factors that are associated with service uptake in a setting, where health services are widely available but utilization is limited, are understudied.

In this context, personality traits may be an additional important but underestimated patient-related determinant of health care utilization in the era of person-centered and evidence-based medicine [12]. Literature identified five personality characteristics that are associated with use of preventive health care use [13]: conscientious (individuals tend to be careful and/or diligent and are usually task and goal-directed), neuroticism (individuals tend to feel depressed, anxious, impulsive, and display angry hostility), openness to experience (individuals being imaginative and are open for new ideas), extraversion (individuals are sociable and outgoing), and lastly agreeableness (individuals are compliant, tend to be altruistic and warm). Yet, the association between personality factors and use of dental services remains largely unexplored [14–16].

Screening in disease prevention and treatment is one of the priorities of health promotion. So far, disease prevention and early detection is only possible, when health services are regularly used by the public.

Worldwide prevalent oral diseases are no exception, thus, the present study will investigate the association between the Big Five personality traits and regularity of lifetime dental visit attendance, while taking demographic and socioeconomic factors into consideration, in a nation-representative older adult population from Germany.

## Materials and methods

### Sample

A cross-sectional study was conducted on available German data from wave 7 (taking place in the year 2017) of the Survey of Health Ageing and Retirement in Europe (SHARE) [17]. We restricted our main analyses to Germany due to differences in the healthcare system between the countries. SHARE includes participants residing in private households aged 50 years and above and their spouses in various European countries and Israel. Health status, demographic, socioeconomic, and social factors are collected via computer-assisted and/or paper-based surveys with personal interviews in SHARE and kept as nationally representative samples. Further details of the study and its protocol can be found in the publication by Börsch-Supan et al. from 2013 [18]. Ethical approval of SHARE from first to fourth waves was obtained from the Ethics Committee of the University of Mannheim. In 2018, the Ethics Council of the Max Planck-Society reviewed and approved the fourth and the consecutive waves of the SHARE project. Oral consent was given by SHARE participants prior to the CAPI interview.

### Assessment of regular and irregular dental visits

The primary outcome measure was the regular dental visit attendance, which was recorded as binary categorical variable. Participants were asked the following question and requested to answer with ‘yes’ or ‘no’: “Now consider your entire life. Have you ever gone to a dentist regularly for check-ups or dental care?”. Participants that answered with a ‘yes’ were classified as regulars and with ‘no’ were classified as irregulars.

### Assessment of personality traits

Personality factors were the main independent variables. The 10-item Big Five Inventory (BFI-10; self-rating scale) [19] was used to assess personality (in terms of agreeableness, conscientiousness, extraversion, neuroticism and openness to experience). It is an established personality inventory measuring the Big Five personality dimensions (with two items each; each dimension ranges from 1 to 5; where a higher value represents a stronger correspondent trait). Personality trait was included as the main independent variable in the regression analyses.

### Covariates

In accordance with previous research [14], covariates were selected. In directed acyclic graphs (DAG) notation, our aim was to block the backdoor path (i.e., a non-causal path from *X* to *Y*; usually common causes of *X* and *Y* [20]) between personality factors and regular dental visit attendance via sociodemographic and health-related factors. Age (continuous), sex (female; male), marital status (registered partnership/married and living together with spouse; married, living separated from spouse; never married; divorced; widowed), born in Germany (no; yes), employment status (retired; employed/self-employed; unemployed; permanently sick or disabled; homemaker; other), education (primary education; secondary education; tertiary education; according to the International Standard Classification of Education (ISCED)-97 [21]), and obesity (body-mass-index ≥ 30 kg/m^2^; using self-rated height and weight) were included as covariates. Moreover, a chronic conditions count (sum score from 0 to 11: high blood pressure or hypertension; high blood cholesterol; stroke or cerebral vascular disease; diabetes or high blood sugar; chronic lung disease; arthritis, including osteoarthritis, or rheumatism; cancer or malignant tumor; stomach or duodenal ulcer, peptic ulcer; Parkinson’s disease; cataracts; hip fracture or femoral fracture) was used as covariate.

### Statistical analyses

The sample characteristics are shown stratified by regular dental visits. Moreover, multiple logistic regressions were used to investigate the association between personality factors and regular dental visits. The results from the logistic regression analyses were reported as odds ratios with 95% confidence intervals and *p* values. The significance level was set at 0.05. All statistical analyses were performed using Stata 16.0 (Stata Corp., College Station, Texas).

## Results

### Descriptive statistics

The mean age of the participants was 66.6 years (SD 9.2 years; 50–95 years) and 52% of the participants were females in the total analytical sample. About 84% of the individuals had reported to have attended regular dental visits during lifetime. Stratified by regular dental visits, sample characteristics are shown in Table 1. Significant differences between these groups were present in age, sex, country of origin, educational level, employment status, chronic conditions, conscientiousness, extraversion, and openness to experience.

**Table 1:** Sample characteristics for the analytical sample (stratified by regular dental visits; *n* = 2822)

	Not regular dental visits	Regular dental visits	*p* value
	(*n* = 453)	(*n* = 2369)	
	Mean (SD)/*n* (%)	Mean (SD)/*n* (%)	
Age (in years)	68.8 (9.7)	66.2 (9.0)	< .001
Sex			.001
Men	273 (20.2%)	1080 (79.8%)	
Women	180 (12.3%)	1289 (87.7%)	
Country of origin			< .001
Not born in country of interview	83 (24.1%)	262 (75.9%)	
Born in country of interview	370 (14.9%)	2107 (85.1%)	
Marital status			.60
Married, living separated from spouse; never married; divorced; widowed	158 (20.4%)	615 (79.6%)	
Married and living together with spouse; registered partnership	295 (14.4%)	1754 (85.6%)	
Education			< .001
Primary education	98 (32.6%)	203 (67.4%)	
Secondary education	266 (16.5%)	1351 (83.5%)	
Tertiary education	89 (9.8%)	815 (90.2%)	
Employment status			< .001
Retired	276 (17.8%)	1276 (82.2%)	
Employed/self-employed	111 (11.7%)	839 (88.3%)	
Unemployed	11 (19.0%)	47 (81.0%)	
Permanently sick or disabled	17 (19.5%)	70 (80.5%)	
Homemaker	31 (20.5%)	120 (79.5%)	
Other	7 (29.2%)	17 (70.8%)	
Weight category			.21
Non-obese	332 (15.6%)	1797 (84.4%)	
Obese	121 (17.5%)	572 (82.5%)	
Chronic conditions (from 0 to 11)	1.5 (1.4)	1.2 (1.2)	< .001
Agreeableness (from 1 to 5, higher values reflect higher agreeableness)	3.4 (0.9)	3.4 (0.8)	.12
Conscientiousness (from 1 to 5, higher values reflect higher conscientiousness)	4.0 (0.9)	4.2 (0.8)	< .001
Extraversion (from 1 to 5, higher values reflect higher extraversion)	3.2 (1.0)	3.5 (1.0)	< .001
Neuroticism (from 1 to 5, higher values reflect higher neuroticism)	2.6 (1.0)	2.6 (1.0)	.43
Openness to experience (from 1 to 5, higher values reflect higher openness to experience)	3.2 (1.0)	3.5 (1.0)	< .001

With regard to effect sizes (Cohen’s *d*), the association between agreeableness and regular dental visits during lifetime was 0.10 (in parentheses: among all individuals included in the SHARE study: 0.12). Cohen’s *d* for the association between conscientiousness and regular dental visits during lifetime was 0.27 (0.06). Cohen’s *d* for the association between extraversion and regular dental visits during lifetime was 0.21 (0.11). Cohen’s *d* for the association between neuroticism and regular dental visits during lifetime was − 0.06 (− 0.15). Cohen’s *d* for the association between openness to experience and regular dental visits during lifetime was 0.28 (0.25).

### Association between regularity of lifetime dental visit attendance and the Big Five personality traits

In unadjusted logistic regressions (not shown), 2915 individuals were included. After adjusting for sociodemographic factors (age, sex, marital status, born in Germany, employment status, and education), 2864 individuals were included. When obesity and a chronic conditions count were also added to regressions, 2822 individuals remained in the analytical sample.

Detailed results from multiple logistic regression analyses are given in Table 2. Regressions showed that the likelihood of regular dental visits was positively associated with increased extraversion [OR 1.13 (95% CI 1.01–1.26)], increased conscientiousness [OR 1.26 (95% CI 1.10–1.44)], and increased openness to experience [OR 1.12 (95% CI 1.01–1.26). However, regular dental visit attendance was not associated with agreeableness and neuroticism. Moreover, while the outcome measure was positively associated with younger age, being female, born in Germany, being married, higher education, being retired (compared to being homemaker), it was not associated with obesity or chronic diseases.

**Table 2:** Determinants of regular dental visits (0 = no regular dental visits, 1 = regular dental visits)

Independent variables	Regular dental visits
Age	0.98** (0.96–0.99)
Sex: women (reference category: men)	2.27*** (1.78–2.89)
Country of origin: born in country of interview (reference category: not born in country of interview)	1.60** (1.20–2.15)
Marital status: married and living together with spouse; registered partnership (reference category: other^†^)	1.45** (1.14–1.83)
Education: secondary education (reference category: primary education)	2.19*** (1.62–2.98)
Tertiary education	4.40*** (3.04–6.37)
Employment status:—Employed/self-employed (Reference category: retired)	0.92 (0.66–1.30)
Unemployed	0.88 (0.41–1.87)
Permanently sick or disabled	0.88 (0.47–1.66)
Homemaker	0.55* (0.34–0.89)
Other	0.40^+^ (0.15–1.04)
Weight category: obesity (ref.: non-obesity)	1.03 (0.80–1.32)
Chronic conditions (count score)	0.93 (0.86–1.02)
Agreeableness (from 1 to 5, higher values reflect higher agreeableness)	1.09 (0.96–1.24)
Conscientiousness (from 1 to 5, higher values reflect higher conscientiousness)	1.26*** (1.10–1.44)
Extraversion (from 1 to 5, higher values reflect higher extraversion)	1.13* (1.01–1.26)
Neuroticism (from 1 to 5, higher values reflect higher neuroticism)	0.94 (0.84–1.05)
Openness to experience (from 1 to 5, higher values reflect higher openness to experience)	1.12* (1.01–1.26)
Constant	36.32*** (1.73)
Observations	2822
Pseudo *R*^2^	0.09

Findings of multiple logistic regressions. Odds ratios were reported; 95% CI in parentheses

*p < 0.05

**p < 0.01

***p < 0.001

^+^*p* < 0.10

^†^Other including: married, living separated from spouse; never married; divorced; widowed

In sensitivity analysis, rather implausible BMI values ≥ 50 kg/m^2^ were removed. The main findings remained nearly the same in terms of significance and effect size (see Supplementary Table 1).

In further sensitivity analysis, regressions were stratified by age group (50 to 64 years; 65 to 74 years; 75 years and over; see Supplementary Table 2). In this regression analysis, particularly conscientiousness was associated with the likelihood of regular dental visits among individuals aged 50 to 64 years [OR 1.43 (95% CI 1.15–1.79)] and among individuals aged 75 years and over [OR 1.45 (95% CI 1.11–1.88)].

In a last analysis, regressions were no longer restricted to Germany (see Supplementary Table 3). The likelihood of regular dental visits was positively associated with increased extraversion [OR 1.05 (95% CI 1.03–1.07)], increased agreeableness [OR 1.12 (95% CI 1.09–1.14)], decreased neuroticism [OR 0.91 (95% CI 0.89–0.93)] and increased openness to experience [OR 1.14 (95% CI 1.12–1.17) among all older Europeans.

## Discussion

Using nationally representative data, our study showed that the likelihood of regular lifetime dental visit attendance was positively associated with increased extraversion, increased conscientiousness, and increased openness to experience, whereas the outcome measure was not associated with agreeableness and neuroticism. Furthermore, the outcome measure was positively associated with younger age, being female, born in Germany, being married, higher education, being retired (compared to being homemaker), whereas it was not associated with obesity or chronic diseases.

A number of studies suggest that higher scores of conscientiousness are significant predictors of better compliance in periodontal maintenance therapy [22]. Conscientious individuals tend to be more efficient and well-organized. Usually they are more task and goal-directed and the association to longtime regular dental visits appears plausible. A systematic review reported an association between extraversion and conscientiousness with increased uptake of cancer screening [23]. This is in line with our findings and it is suspected, that individuals with higher scores in extraversion are in general more sociable and energetic, thus having a more positive attitude towards their dental appointments. Lastly, different behavioral patterns might explain the lack of dental visits in neurotic individuals: dental anxiety can arise in neurotic individuals [24] and can result in maladaptive behavior.

Furthermore, previous studies suggested that personality traits might be related to treatment adherence and compliance [25, 26], treatment medication adherence [27, 28], and doctor visits in general [29, 30], which in certain conditions significantly reduce quality of life [29], such as in rheumatoid arthritis [31, 32], depression [33, 34], attention deficit hyperactivity disorder [35], multiple sclerosis [36], and cancer screening [23]. Oral diseases are also a significant contributor to reduced quality of life [37]. Thus, increasing uptake of regular dental visits is important to prevent and treat oral diseases and improve quality of life.

This is the first study examining the association between personality characteristics and regular dental visits in Germany. Identification of subjective indicators that are associated with healthcare utilization may complement interventions and/or treatments to increase attendance, compliance and maintenance for individuals at risk for irregular dental visits, and thus improve oral health. Based on the results, one way to increase the attendance to regular dental visits, and thus screening and prevention for oral diseases, could be the improvement of health literacy through personalized education programs addressed to specific personality types [38]. Next to important and well-known socioeconomic and demographic factors, also personality should be considered for the patient-centered oral health care planning.

### Strengths and limitations

Data were taken from the nationally representative SHARE study. The BFI-10 was used to quantify personality factors. One question with a high face validity was used to quantify the outcome measure. It should be noted that the SHARE study has a small sample selection bias [10] and longitudinal studies are required to confirm our results based on cross-sectional data.

## Supplementary Information

Below is the link to the electronic supplementary material.

Supplementary file1 (DOCX 32 KB)
