# Supplementary material for: The Big Five personality traits and regularity of lifetime dental visit attendance: evidence of the Survey of Health, Ageing, and Retirement in Europe (SHARE)
Source: Aging Clin Exp Res. 2021 Dec 28;34(6):1439–45. doi: 10.1007/s40520-021-02051-2 (PMC9151578; doi:10.1007/s40520-021-02051-2)
Supplement: Supplementary file 1 — Supplementary file1 (DOCX 32 KB) [file 40520_2021_2051_MOESM1_ESM.docx]

Supplementary Table 1. Determinants of regular dental visits (0 = no regular dental visits, 1 = regular dental visits). Findings of multiple logistic regressions (BMI values ≥ 50 kg/m² were removed)

| Independent variables | Regular dental visits |
| --- | --- |
|  |  |
| Age | 0.97** |
|  | (0.96 - 0.99) |
| Sex: Women (Reference category: Men) | 2.35*** |
|  | (1.85 - 3.00) |
| Country of origin: Born in country of interview (Reference category: Not born in country of interview) | 1.59** |
|  | (1.19 - 2.13) |
| Marital status: Married and living together with spouse; registered partnership (Reference category: Other†) | 1.44** |
|  | (1.14 - 1.82) |
| Education: Secondary education (Reference category: Primary education) | 2.22*** |
|  | (1.64 - 3.01) |
| - Tertiary education | 4.44*** |
|  | (3.08 - 6.42) |
| Employment status: - Employed/self-employed (Reference category: retired) | 0.96 |
|  | (0.68 - 1.35) |
| - Unemployed | 0.88 |
|  | (0.42 - 1.88) |
| - Permanently sick or disabled | 0.80 |
|  | (0.42 - 1.50) |
| - Homemaker | 0.55* |
|  | (0.34 - 0.89) |
| - Other | 0.46 |
|  | (0.17 - 1.26) |
| Weight category: Obesity (Ref.: Non-obesity) | 0.99 |
|  | (0.78 - 1.27) |
| Chronic conditions (count score) | 1.09 |
|  | (0.96 - 1.24) |
| Agreeableness (from 1 to 5, higher values reflect higher agreeableness) | 1.23** |
|  | (1.07 - 1.41) |
| Conscientiousness (from 1 to 5, higher values reflect higher conscientiousness) | 1.13* |
|  | (1.01 - 1.27) |
| Extraversion (from 1 to 5, higher values reflect higher extraversion) | 0.93 |
|  | (0.83 - 1.04) |
| Neuroticism (from 1 to 5, higher values reflect higher neuroticism) | 1.14* |
|  | (1.02 - 1.27) |
| Openness to experience (from 1 to 5, higher values reflect higher openness to experience) | 0.82 |
|  | (0.18 - 3.76) |
| Constant | 0.97** |
|  | (0.96 - 0.99) |
|  |  |
| Observations | 2,821 |
| Pseudo R² | 0.09 |

Odds ratios were reported; 95% CI in parentheses; *** p<0.001, ** p<0.01, * p<0.05, + p<0.10; † Other including: Married, living separated from spouse; never married; divorced; widowed

Supplementary Table 2. Determinants of regular dental visits (0 = no regular dental visits, 1 = regular dental visits). Findings of multiple logistic regressions (stratified by age group)

| Independent variables | Regular dental visits – among individuals 50 to 64 years | Regular dental visits – among individuals 65 to 74 years | Regular dental visits – among individuals 75 years and over |
| --- | --- | --- | --- |
|  |  |  |  |
| Age | 1.02 | 1.00 | 0.99 |
|  | (0.96 - 1.09) | (0.93 - 1.07) | (0.94 - 1.04) |
| Sex: Women (Reference category: Men) | 2.24*** | 3.23*** | 1.69* |
|  | (1.51 - 3.32) | (2.05 - 5.08) | (1.05 - 2.70) |
| Country of origin: Born in country of interview (Reference category: Not born in country of interview) | 2.42*** | 1.78* | 0.88 |
|  | (1.47 - 3.98) | (1.07 - 2.96) | (0.51 - 1.53) |
| Marital status: Married and living together with spouse; registered partnership (Reference category: Other†) | 1.41+ | 1.84** | 1.33 |
|  | (0.95 - 2.10) | (1.19 - 2.85) | (0.85 - 2.08) |
| Education: Secondary education (Reference category: Primary education) | 2.86*** | 1.59 | 2.28** |
|  | (1.68 - 4.86) | (0.86 - 2.92) | (1.34 - 3.88) |
| - Tertiary education | 5.06*** | 4.71*** | 3.72*** |
|  | (2.69 - 9.52) | (2.25 - 9.83) | (1.91 - 7.25) |
| Employment status: - Employed/self-employed (Reference category: retired) | 0.79 | 0.94 | 0.24 |
|  | (0.41 - 1.51) | (0.39 - 2.29) | (0.02 - 2.90) |
| - Unemployed |  |  |  |
|  |  |  |  |
| - Permanently sick or disabled |  |  |  |
|  |  |  |  |
| - Homemaker | 0.40* | 0.40+ | 0.79 |
|  | (0.17 - 0.97) | (0.14 - 1.12) | (0.32 - 1.92) |
| - Other |  |  |  |
|  |  |  |  |
| Weight category: Obesity (Ref.: Non-obesity) | 0.37 | 0.18 |  |
|  | (0.11 - 1.29) | (0.01 - 3.32) |  |
| Chronic conditions (count score) | 1.02 | 0.96 | 1.07 |
|  | (0.68 - 1.54) | (0.62 - 1.48) | (0.63 - 1.81) |
| Agreeableness (from 1 to 5, higher values reflect higher agreeableness) | 0.91 | 0.90 | 0.99 |
|  | (0.77 - 1.07) | (0.77 - 1.04) | (0.85 - 1.14) |
| Conscientiousness (from 1 to 5, higher values reflect higher conscientiousness) | 1.24+ | 0.91 | 1.11 |
|  | (1.00 - 1.53) | (0.72 - 1.14) | (0.88 - 1.40) |
| Extraversion (from 1 to 5, higher values reflect higher extraversion) | 1.43** | 0.93 | 1.45** |
|  | (1.15 - 1.79) | (0.73 - 1.20) | (1.11 - 1.88) |
| Neuroticism (from 1 to 5, higher values reflect higher neuroticism) | 1.17+ | 1.04 | 1.30* |
|  | (0.97 - 1.42) | (0.85 - 1.27) | (1.05 - 1.61) |
| Openness to experience (from 1 to 5, higher values reflect higher openness to experience) | 0.98 | 0.96 | 0.82+ |
|  | (0.81 - 1.19) | (0.78 - 1.17) | (0.66 - 1.02) |
| Constant | 1.04 | 1.12 | 1.14 |
|  | (0.87 - 1.25) | (0.92 - 1.37) | (0.92 - 1.41) |
|  |  |  |  |
| Observations |  |  |  |
| Pseudo R² |  |  |  |

Odds ratios were reported; 95% CI in parentheses; *** p<0.001, ** p<0.01, * p<0.05, + p<0.10; † Other including: Married, living separated from spouse; never married; divorced; widowed. Please note that some variables (regarding employment status) were dropped.

Supplementary Table 3. Determinants of regular dental visits (0 = no regular dental visits, 1 = regular dental visits). Findings of multiple logistic regressions (among all older Europeans; thus, not restricted to Germany anymore)

| Independent variables | Regular dental visits |
| --- | --- |
|  |  |
| Age | 0.99*** |
|  | (0.98 - 0.99) |
| Sex: Women (Reference category: Men) | 1.77*** |
|  | (1.70 - 1.85) |
| Country of origin: Born in country of interview (Reference category: Not born in country of interview) | 0.97 |
|  | (0.91 - 1.04) |
| Marital status: Married and living together with spouse; registered partnership (Reference category: Other†) | 1.05* |
|  | (1.01 - 1.10) |
| Education: Secondary education (Reference category: Primary education) | 2.00*** |
|  | (1.91 - 2.09) |
| - Tertiary education | 4.02*** |
|  | (3.78 - 4.29) |
| Employment status: - Employed/self-employed (Reference category: retired) | 1.16*** |
|  | (1.09 - 1.23) |
| - Unemployed | 0.72*** |
|  | (0.64 - 0.82) |
| - Permanently sick or disabled | 0.77*** |
|  | (0.69 - 0.86) |
| - Homemaker | 0.53*** |
|  | (0.49 - 0.58) |
| - Other | 0.64*** |
|  | (0.55 - 0.75) |
| Weight category: Obesity (Ref.: Non-obesity) | 0.89*** |
|  | (0.85 - 0.93) |
| Chronic conditions (count score) | 1.00 |
|  | (0.99 - 1.02) |
| Agreeableness (from 1 to 5, higher values reflect higher agreeableness) | 1.12*** |
|  | (1.09 - 1.14) |
| Conscientiousness (from 1 to 5, higher values reflect higher conscientiousness) | 1.02 |
|  | (0.99 - 1.04) |
| Extraversion (from 1 to 5, higher values reflect higher extraversion) | 1.05*** |
|  | (1.03 - 1.07) |
| Neuroticism (from 1 to 5, higher values reflect higher neuroticism) | 0.91*** |
|  | (0.89 - 0.93) |
| Openness to experience (from 1 to 5, higher values reflect higher openness to experience) | 1.14*** |
|  | (1.12 - 1.17) |
| Constant | 1.39* |
|  | (1.05 - 1.84) |
|  |  |
| Observations | 56,221 |
| Pseudo R² | 0.08 |

Odds ratios were reported; 95% CI in parentheses; *** p<0.001, ** p<0.01, * p<0.05, + p<0.10; † Other including: Married, living separated from spouse; never married; divorced; widowed
